# α‐Synuclein species as potential cerebrospinal fluid biomarkers for dementia with lewy bodies

**DOI:** 10.1002/mds.111

**Published:** 2018-11-15

**Authors:** Inger van Steenoven, Nour K. Majbour, Nishant N. Vaikath, Henk W. Berendse, Wiesje M. van der Flier, Wilma D.J. van de Berg, Charlotte E. Teunissen, Afina W. Lemstra, Omar M.A. El‐Agnaf

**Affiliations:** ^1^ Department of Neurology and Alzheimer Center, Amsterdam Neuroscience VU University Medical Center Amsterdam The Netherlands; ^2^ Neurological Disorders Research Center, Qatar Biomedical Research Institute (QBRI) Hamad Bin Khalifa University (HBKU), Qatar Foundation Doha Qatar; ^3^ Department of Epidemiology and Biostatistics VU University Medical Center Amsterdam The Netherlands; ^4^ Department of Anatomy and Neurosciences, section Clinical Neuroanatomy, Amsterdam Neuroscience VU University Medical Center Amsterdam The Netherlands; ^5^ Neurochemistry Laboratory and Biobank, Department of Clinical Chemistry, Amsterdam Neuroscience VU University Medical Center Amsterdam The Netherlands; ^6^ Life Sciences Division, College of Science and Engineering, Hamad Bin Khalifa University (HBKU), Education City, Qatar Foundation Doha Qatar

**Keywords:** biomarkers, CSF, α‐synuclein, dementia with Lewy bodies

## Abstract

**Background:** The objective of this study was to investigate the discriminating value of a range of CSF α‐synuclein species for dementia with Lewy bodies compared with Alzheimer's disease, PD, and cognitively normal controls.

**Methods:** We applied our recently published enzyme‐linked immunosorbent assays to measure the CSF levels of total α‐synuclein, oligomeric α‐synuclein, and phosphorylated α‐synuclein in dementia with Lewy bodies (n = 42), Alzheimer's disease (n = 39), PD (n = 46), and controls (n = 78). General linear models corrected for age and sex were performed to assess differences in α‐synuclein levels between groups. We used backward‐elimination logistic regression analysis to investigate the combined discriminating value of the different CSF α‐synuclein species and Alzheimer's disease biomarkers.

**Results:** CSF levels of total α‐synuclein were lower in dementia with Lewy bodies and PD compared with Alzheimer's disease as well as controls (*P* < 0.001). In contrast, CSF levels of oligomeric α‐synuclein were higher in dementia with Lewy bodies and PD compared with Alzheimer's disease (*P* < 0.05) and controls (*P* < 0.001). No group differences were found for phosphorylated α‐synuclein. In dementia with Lewy bodies and PD, CSF total α‐synuclein levels positively correlated with tau and phosphorylated tau (both *r* > 0.40, *P* < 0.01), but not with amyloid‐β_1‐42_. The optimal combination to differentiate dementia with Lewy bodies from controls consisted of amyloid‐β_1‐42_, tau, total α‐synuclein, oligomeric α‐synuclein, age, and sex (AUC, 0.90). To differentiate dementia with Lewy bodies from Alzheimer's disease, the combination of tau and oligomeric α‐synuclein resulted in an AUC of 0.83. CSF α‐synuclein species do not contribute to the differentiation of dementia with Lewy bodies from PD.

**Conclusions:** CSF α‐synuclein species could be useful as part of a biomarker panel for dementia with Lewy bodies. Evaluating both oligomeric α‐synuclein and total α‐synuclein in CSF helps in the diagnosis of dementia with Lewy bodies. © 2018 The Authors. *Movement Disorders* published by Wiley Periodicals, Inc. on behalf of International Parkinson and Movement Disorder Society.

Dementia with Lewy bodies (DLB) is the second most common form of dementia in people older than 65 years. It accounts for 10%‐20% of dementia cases.^1^ DLB is characterized by cognitive decline in combination with visual hallucinations, fluctuating cognition, and parkinsonism, as well as Rapid eye movement (REM) sleep behavior discorder (RBD) autonomic dysfunction.^2^ Because of heterogeneity in clinical presentation and clinical and pathological overlap between DLB, Parkinson's disease (PD), and Alzheimer's disease (AD), accurate diagnosis of DLB is often challenging, especially in early stages of the disease.^3^ Currently, the diagnosis of DLB is based on clinical diagnostic consensus criteria.^2^, ^4^ These diagnostic criteria have a high specificity (80%‐100%) but low sensitivity (20%‐60%). As a consequence, more than 80% of DLB cases are initially diagnosed with other disorders, mainly AD or PD.^5^ Postmortem pathological confirmation of the presence of cortical Lewy bodies and Lewy neurites — intraneuronal inclusions primarily composed of α‐synuclein (α‐syn) aggregates^6^ — constitutes the diagnostic gold standard. However, accurate diagnosis antemortem is important for adequate clinical management and patient care. There is an urgent need to discover biomarkers that can aid in an accurate and early diagnosis of DLB.

Analysis of cerebrospinal fluid (CSF) biomarkers is increasingly applied in the diagnostic workup of neurodegenerative diseases. CSF amyloid‐β_1‐42_ (Aβ_1‐42_), total tau protein (t‐tau), and phosphorylated tau at threonine 181 (p‐tau) mirror the main neuropathological hallmarks of AD and are well established to aid in the diagnosis of AD.^7^ AD‐like pathology, that is, neurofibrillary tangles and amyloid plaques, is also found in almost half of patients with DLB.^8^, ^9^ Therefore, CSF AD biomarkers have an added value to distinguish DLB from healthy subjects and to some extent from PD. However, to distinguish DLB from AD, additional biomarkers are necessary.

The discovery of α‐syn as a major component of Lewy bodies^10^ and the detection of α‐syn in CSF^11^, ^12^ have encouraged research into α‐syn as a potential CSF biomarker for both DLB and PD. The discriminating value of CSF total α‐syn (t‐α‐syn) has been addressed in multiple studies, with conflicting results. Although some studies have shown that CSF levels of t‐α‐syn are decreased in patients with PD, Parkinson's disease dementia (PDD) or DLB compared with controls or patients with AD, other studies demonstrated increased levels or no group differences at all (see references 13, 14, 15 for review). These mixed results could be a result of a number of methodological factors, such as use of different antibodies and standard proteins in the immunoassays, patient selection, variation in preanalytical processing, and blood contamination from traumatic lumbar puncture.^13^ Moreover, previous studies all used immunoassays that detect CSF t‐α‐syn, not taking into account its conformation or aggregation state, and thus CSF t‐α‐syn might lack disease specificity.

Soluble α‐syn oligomers (o‐α‐syn) could be more useful, because (1) early aggregates, or “soluble oligomers”, of α‐syn might play a more essential role in the pathogenesis of α‐synucleinopathies rather than the late aggregates; (2) oligomeric forms of α‐syn seem to be neurotoxic/more pathogenic in vitro and in vivo^6^, ^16^, ^17^; and (3) soluble α‐synuclein oligomers have been linked to synaptic and neuronal degeneration in an α‐syn E57K transgenic mouse model.^18^ Postmortem studies have shown high levels of soluble o‐α‐syn in the brains of patients with PD and DLB compared with patients with AD and controls.^19^, ^20^ Another α‐syn species of interest is α‐syn phosphorylated at serine 129 (pSer129‐α‐syn). pSer129‐α‐syn is specifically associated with Lewy body pathology because approximately 90% of accumulated α‐syn in Lewy bodies consists of pSer129‐α‐syn.^21^ To investigate the use of CSF α‐syn species as biomarkers for the diagnosis of DLB, we recently developed robust and specific enzyme‐linked immunosorbent assays (ELISAs) to quantify a wide range of α‐syn species (t‐α‐syn, o‐α‐syn, and pSer129‐α‐syn) in CSF.^20^ We and others reported increased levels of soluble o‐α‐syn in PD and PDD patients compared with patients with other neurological disorders^22^, ^23^, ^24^, ^25^ and healthy controls.^20^, ^26^ Only one study showed that soluble o‐α‐syn was increased in DLB patients compared with patients with AD, but not compared with controls.^22^ Two recent CSF studies reported elevated pSer129‐α‐syn levels in the CSF of PD patients compared with controls.^20^, ^27^ No studies investigating levels of CSF pSer129‐α‐syn in patients with DLB have been published yet.

The aim of this study was to assess the diagnostic value of measuring CSF levels of a wide range of different CSF α‐syn species (t‐α‐syn, o‐α‐syn, and pSer129‐α‐syn) for the diagnosis of DLB in a well‐established cohort of DLB patients, PD patients, AD patients, and cognitively normal controls using our recently developed assays. In addition, we investigated whether these CSF α‐syn species add discriminatory value to the CSF AD biomarkers.

## Methods

### Participants

We included 106 participants with available CSF from the Amsterdam Dementia Cohort who had visited the VUmc Alzheimer Center between 2002 and 2015 (41 DLB patients, 35 AD patients, and 30 controls with subjective cognitive decline [SCD]). Patients with AD and with SCD were matched for age and sex with DLB patients. In addition, data and CSF samples of 46 PD patients and 48 volunteers without neurological symptoms collected for a previous study^20^ at the VUmc outpatient clinic for movement disorders were also included in our analyses.

The study was conducted according to the revised Declaration of Helsinki and Good Clinical Practice guidelines and approved by the local ethics committee of the VU University Medical Center. All study participants gave written informed consent for use of their clinical data and biomaterial for research purposes.

### Clinical Diagnosis

All patients received a standardized and multidisciplinary workup, including medical history, physical, neurological, and neuropsychological examination, MRI, and laboratory tests. Diagnoses were made in multidisciplinary consensus meetings without knowledge of CSF AD biomarker results.^28^, ^29^

DLB patients were diagnosed according to the 2005 consensus criteria for probable DLB^4^ and also fulfilled novel consensus criteria.^2^ The diagnosis of DLB was supported by ^123^I–FP‐CIT single‐photon emission computed tomography (SPECT) findings showing presynaptic dopaminergic deficits (n = 32) or slow‐wave activity on electroencephalogram (EEG; n = 8) or was confirmed at autopsy (n = 1). AD patients were diagnosed using the criteria of the National Institute for Neurological and Communicative Diseases AD and Related Disorders Association criteria for probable AD.^30^ PD patients were diagnosed according to the United Kingdom PD Society Brain Bank clinical diagnostic criteria by movement disorders specialists.^31^ The diagnosis of PD was supported by abnormal ^123^I–FP‐CIT SPECT scans (n = 21). Severity of parkinsonism in the “on” state was evaluated using the UPDRS‐III. PD patients were only included if the MMSE and/or neuropsychological assessments did not indicate dementia. Subjects were labeled as SCD when the cognitive complaints could not be confirmed by cognitive testing, and criteria for mild cognitive impairment, dementia or any other neurological or psychiatric disorder known to cause cognitive complaints were not met. To be included as controls in the present study, SCD subjects had to remain cognitively stable for at least 2 years. Cognition at baseline and yearly follow‐up was evaluated with extensive neuropsychological assessment. Healthy volunteers underwent a standardized clinical assessment that included medical history and neurological examination. Cognitive impairment in the healthy volunteer group was excluded using the Cambridge Cognitive Examination scale. SCD subjects and healthy volunteers were analyzed as a single cognitively normal group (Supplementary Table 1).

### CSF Collection

CSF was obtained by lumbar puncture between the L3/L4 or L4/L5 or L5/S1 intervertebral space using a 25‐gauge needle and syringe, collected in polypropylene tubes, centrifuged at 1800*g* at 4°C for 10 minutes, aliquoted in polypropylene tubes of 0.5 mL, and stored at ‐80°C until further analysis, in line with international guidelines.^32^ A small amount of CSF was used for routine analysis, including total cells, total protein, glucose, and erythrocytes. Only samples containing < 500 erythrocytes per microliter were included in the analysis, as excessive erythrocytes may influence α‐syn levels.^33^

### CSF Assays

CSF levels of Aβ_1‐42_, t‐tau, and p‐tau were determined with sandwich ELISA (Innotest, Fujirebio, Gent, Belgium), as described previously.^34^

CSF t‐α‐syn, pSer129‐α‐syn, and o‐α‐syn levels were measured using our recently published ELISA assays.^20^ More details on the α‐syn assays are described in the supporting information. All biomarker analyses were carried out blinded to the clinical diagnosis.

### Statistical Analysis

Demographical and clinical characteristics were compared between groups using chi‐square tests, analysis of variance with post hoc Bonferroni tests or Kruskal‐Wallis tests followed by Mann‐Whitney *U* tests, where appropriate.

CSF α‐syn levels below the first quartile minus 3 times the interquartile range (IQR) or above the third quartile plus 3 times the IQR were considered outliers and excluded from subsequent analyses (more details about outliers are presented in Supplementary Table 2). CSF t‐tau and p‐tau levels were log‐transformed to meet assumptions of normally distributed data. Other biomarkers had a normal distribution.

For all CSF biomarkers, differences between diagnostic groups were assessed using general linear models (GLMs) corrected for age and sex with post hoc Bonferroni tests. We examined correlations using bivariate Pearson correlation coefficient within diagnostic groups. We used the Benjamini‐Hochberg procedure to correct for multiple testing. Because of collinearity between t‐tau and p‐tau, only the strongest predictor (t‐tau) was included in the following analyses.

Subsequently, we used stepwise linear discriminant function analysis to assess the accuracy of the combined CSF biomarkers in classification of the four groups. Stepwise linear discriminant function analysis identifies canonical discriminant functions based on combinations of biomarkers which contribute maximally to group separation and evaluate how well these canonical discriminant functions discriminate the diagnostic groups.

Finally, to assess which subsets of CSF biomarkers performed best in distinguishing DLB from AD, PD, and controls, we performed multivariate logistic regression analyses with backward stepwise selection (separate analyses for each comparison). DLB was entered as a reference category and Aβ_1‐42_, t‐tau, t‐α‐syn, o‐α‐syn, pSer129‐α‐syn, age, and sex as predictors. CSF data were Z‐transformed. Therefore, the resulting ORs provide the increased odds per standard deviation increase in biomarker value. For the resulting models, we report AUC, sensitivity, specificity, negative predictive value (NPV), positive predictive value (PPV), and OR (95% CI) of the individual biomarkers. Sensitivity, specificity, NPV, and PPV were calculated using the classification table (probability threshold, 0.5).

All statistical analyses were performed using IBM SPSS software for Mac, version 22.0. A *P* < 0.05 was considered significant.

## Results

Demographical, clinical characteristics and CSF biomarkers levels of the diagnostic groups are presented in Table 1. There was an age difference between groups, as PD patients were younger than AD patients (*P* < 0.05). The sex distribution also varied between the diagnostic groups (*P* < 0.001). Patients with dementia (AD and DLB) had lower MMSE scores compared with controls and patients with PD (*P* < 0.001). The GLM showed differences between diagnostic groups for Aβ_1‐42_, t‐tau, and p‐tau (*P* < 0.05, adjusted for age and sex; Table 1). AD patients had a CSF profile with lower levels of Aβ_1‐42_ and higher levels of t‐tau and p‐tau compared with patients with PD and controls. DLB patients had levels in‐between AD and PD patients, with higher levels of Aβ_1‐42_ and lower levels of tau compared with AD patients and lower levels Aβ_1‐42_ and higher levels of tau compared with PD patients and controls. There were no differences between patients with PD and controls.

**Table 1:** Demographics and CSF biomarkers by diagnostic group

	DLB (n = 41)	PD (n = 46)	AD (n = 35)	Controls (n = 78)
Age (y), mean ± SD	66.5 ± 6.1	62.8 ± 10.1^i^	67.8 ± 6.3	64.4 ± 6.9
Sex (male), n (%)	35 (85.4%)^g^, ^j^	28 (60.9%)^h^	33 (94.3%)^g^	41 (52.6%)
Disease duration (y),^a^ median (IQR)	3 (2‐4)	4 (2‐9)	4 (3‐5)	NA
MMSE,^b^ median (IQR)	23 (19‐26)^g^, ^j^	29 (28‐30)^h^	23 (18‐25)^g^	29 (28‐30)
Aβ_1‐42_ (pg/mL), mean ± SD	695 ± 275^f^, ^i^, ^k^	917 ± 211^c^	486 ± 194^f^	926 ± 266
t‐tau (pg/mL), median (IQR)	325 (224‐431)^g^, ^h^, ^j^	189 (157‐275)^h^	588 (398‐787)^f^	247 (174‐308)
p‐tau (pg/mL), median (IQR)	53 (35‐66)^h^	38 (28‐51)^h^	75.0 (62‐99)^f^	45 (35‐57)
t‐α‐syn (ng/mL),^c^ mean ± SD	1.4 ± 0.4^f^, ^h^	1.4 ± 0.3^f^, ^h^	2.0 ± 0.5	1.8 ± 0.6
o‐α‐syn (pg/mL),^d^ mean ± SD	108 ± 34^f^	120 ± 49^f^, ^h^	89 ± 30	72 ± 37
pSer129‐α‐syn (pg/mL),^e^ mean ± SD	232 ± 79	258 ± 52	220 ± 61	235 ± 54

Data are expressed as mean ± SD, median (IQR), or n (%). Demographical differences between groups were analyzed using analysis of variance with post hoc Bonferroni tests (age), X^2^ tests (sex), and Kruskal‐Wallis with post hoc Mann‐Whitney *U* tests (MMSE, disease duration). Differences in CSF biomarker levels between groups were assessed with a GLM adjusted for age and sex. t‐Tau and p‐tau were log‐transformed, but are presented as raw data.

Aβ_1‐42_, amyloid‐β_1‐42_; AD, Alzheimer's disease; DLB, dementia with Lewy bodies; MMSE, Mini‐Mental State Examination; NA, not applicable; PD, Parkinson's disease; pSer129‐α‐synuclein, phosphorylated α‐synuclein protein at serine 129; p‐tau, tau phosphorylated at threonine 181; o‐α‐syn, oligomeric α‐synuclein; t‐α‐syn, total α‐synuclein; t‐tau, total tau protein.

a AD, n = 35; DLB, n = 40; PD, n = 45.

b Controls, n = 78; AD, n = 34; DLB, n = 40; PD, n = 46.

c Controls, n = 77; AD, n = 34; DLB, n = 41; PD, n = 46.

d Controls, n = 78; AD, n = 35; DLB, n = 41; PD, n = 42.

e Controls, n = 75; AD, n = 33; DLB, n = 38; PD, n = 45.

f *P* < 0.001 compared with controls.

g *P* < 0.05 compared with controls.

h *P* < 0.001 compared with AD.

i *P* < 0.05 compared with AD.

j *P* < 0.001 compared with PD.

k *P* < 0.05 compared with PD.

**Figure 1:**
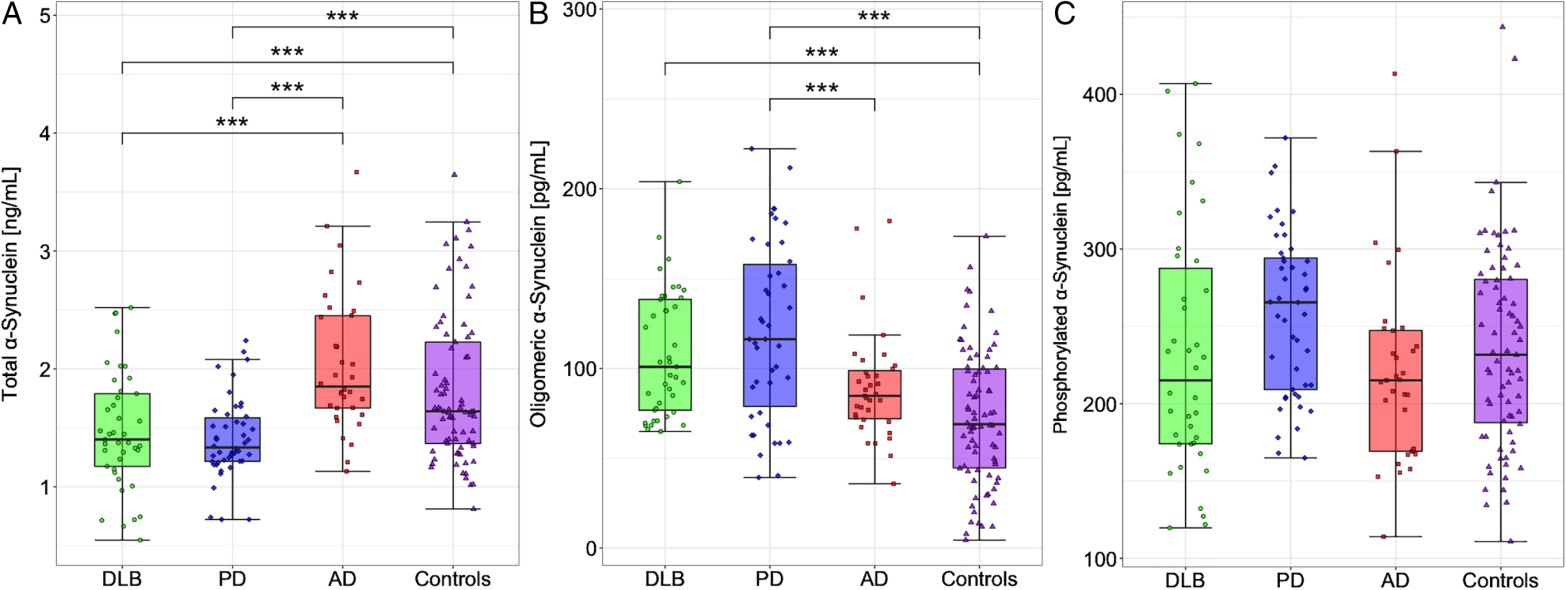
Box‐and‐whiskers plots of CSF levels of α‐syn species in DLB, PD, AD, and controls. (A) CSF levels of t‐α‐syn, (B) CSF levels of o‐α‐syn, (C) CSF levels of pSer129‐α‐syn. The line through the middle of the boxes corresponds to the median and the lower and the upper lines to the 25th and 75th percentiles, respectively. The whiskers extend from the 5th percentile on the bottom to the 95th percentile on top. Differences between groups were assessed with the GLM, adjusted for age and sex. *P < 0.05, **P < 0.01, ***P < 0.001. [Color figure can be viewed at wileyonlinelibrary.com]

The age‐ and sex‐adjusted GLM revealed differences in levels of CSF t‐α‐syn, and o‐α‐syn between groups (both *P* < 0.001; Table 1 and Fig. 1). Subsequent Bonferroni‐adjusted *t* tests showed lower levels of t‐α‐syn in PD and DLB patients compared with AD patients and controls (*P* < 0.001). In contrast, the levels of o‐α‐syn were higher in PD and DLB patients compared with controls (*P* < 0.001). Moreover, o‐α‐syn was also higher in patients with PD compared with patients with AD (*P* < 0.001). There were no group differences for pSer129‐α‐syn. The ratios of o‐α‐syn/t‐α‐syn and pSer129‐α‐syn/t‐α‐syn were both higher in PD and DLB patients compared with the ratios in AD patients and controls (all *P* < 0.01; Supplementary Fig. 1). Analysis including all cases showed similar results (data not shown).

**Figure 2:**
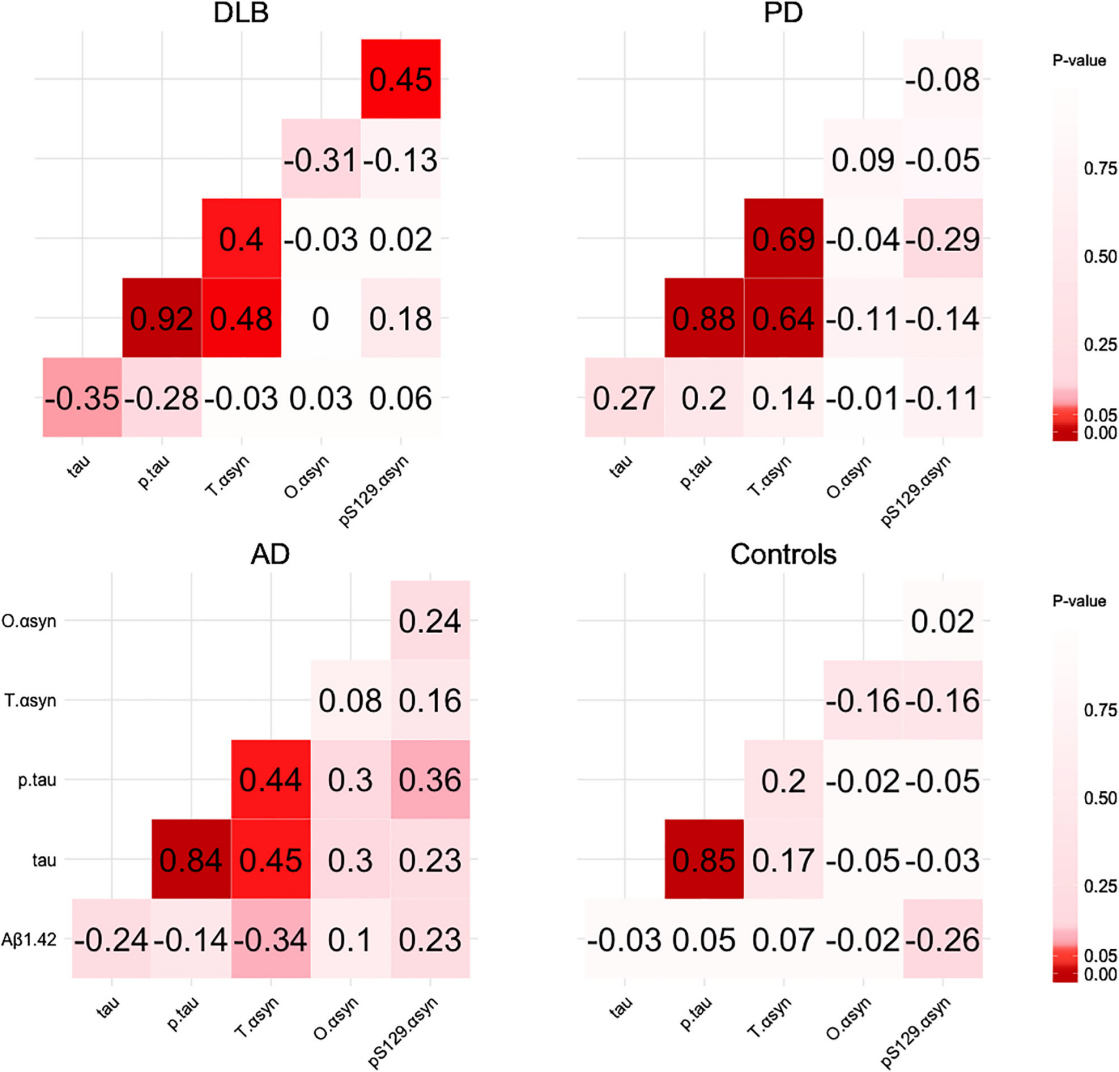
Correlations (Pearson) between CSF biomarkers in the diagnostic groups. Pearson correlation coefficients are depicted by the numbers within the plots. The colors represent the *P* value of the association. Darker colors represent lower *P* values, and lighter colors represent higher *P* values. Aβ_1‐42_, amyloid‐β_1‐42_; AD, Alzheimer's disease; DLB, dementia with Lewy bodies; PD, Parkinson's disease; pS129‐α‐syn, phosphorylated α‐synuclein protein at serine 129; p‐tau, tau phosphorylated at threonine 181; o‐αsyn, α‐synuclein oligomer; t‐α‐syn, total α‐synuclein; t‐tau, total tau protein. [Color figure can be viewed at wileyonlinelibrary.com]

Subsequently, by use of Pearson correlations, we evaluated associations between different CSF biomarkers (Fig. 2, Supplementary Table 3). For DLB, but not for any of the other groups, we found a positive association between o‐α‐syn and pSer129‐α‐syn (*r* = 0.45, *P* < 0.05). Evaluating correlations between α‐syn species and the AD biomarkers, we found a positive correlation between t‐α‐syn and (p)tau in all patient groups (both *r* > 0.40, *P* < 0.05), but not in controls. By contrast, levels of o‐α‐syn and pSer129‐α‐syn did not correlate with any of the AD biomarkers. Correlations of the α‐syn species with clinical parameters (age, disease duration, MMSE, and UPDRS‐III) are shown in Supplementary Table 4. Briefly, we observed a negative correlation between t‐α‐syn and MMSE within the PD group (*r* = ‐0.42, *P* < 0.01), but not in any of the other groups. CSF o‐α‐syn did not correlate with any of the clinical parameters. In the DLB group, we found a positive correlation between pSer129‐α‐syn and age (*r* = 0.39, *P* < 0.05) and a negative correlation between pSer129‐α‐syn and MMSE score (*r* = ‐0.45, *P* < 0.01).

**Figure 3:**
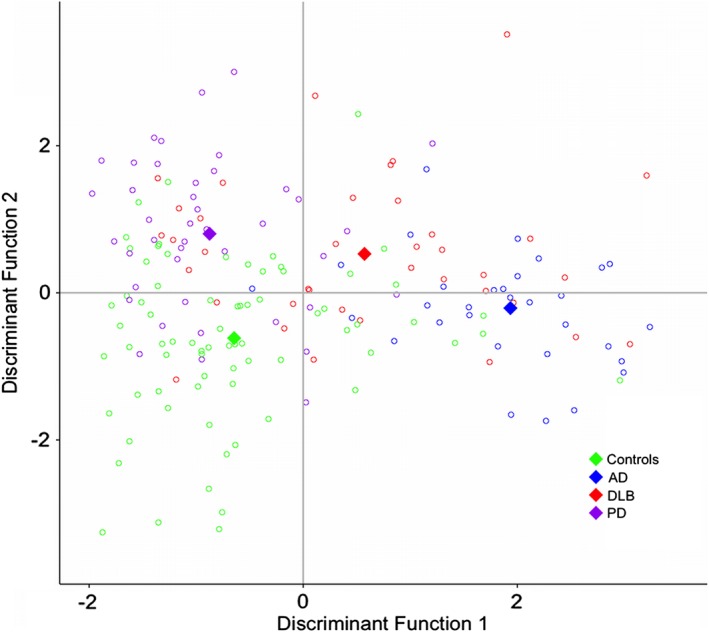
Discriminant function plot of canonical discriminant functions for discrimination of DLB, PD, AD, and controls. Red circles indicate individual data of DLB patients, purple circles indicate individual data of Parkinson's disease patients, blue circles indicate individual data of Alzheimer's disease patients, and green circles indicate individual data of controls. The diamonds represent the group centroids. [Color figure can be viewed at wileyonlinelibrary.com]

Subsequently, we conducted a discriminant analysis to identify the best combination of biomarkers to classify the four groups. A panel of Aβ_1‐42_, t‐tau, t‐α‐syn, and o‐α‐syn together classified 64.5% of all cases correctly in the DLB, AD, PD, and control groups (lambda = 0.351, *P* < 0.001). Figure 3 shows the discrimination plot of the 2 canonical discriminant functions for discrimination of the four groups. The loadings of individual predictors on each discriminant function are shown in Supplementary Table 5. Canonical discriminant function 1 strongly correlates with the AD biomarkers (A_1‐42_, *r* = 0.647; t‐tau, *r* = ‐0.789; p‐tau, *r* = ‐0.596) and discriminated AD patients and DLB patients from PD patients and controls. We will refer to this function as the dementia function. Canonical discriminant function 2 strongly correlates with the α‐syn species (t‐α‐syn, *r* = ‐0.620; o‐α‐syn, *r* = 0.829; pSer129‐α‐syn, *r* = 0.205) and adds by discriminating PD patients and DLB patients from AD patients and controls. We will refer to this function as the movement disorders function. DLB is located at the intersection of both the dementia axis and the movement disorders axis.

Finally, we used backward‐elimination multiple logistic regression analyses to identify optimal biomarker panels for bilateral comparisons between (1) DLB patients and controls, (2) DLB and AD patients, and (3) DLB and PD patients. Aβ_1‐42_, t‐tau, t‐α‐syn, o‐α‐syn, pSer129‐α‐syn, age, and sex were entered as predictors. The DLB group was used as the reference group in each comparison. Table 2 shows a summary of the final models. The combination of Aβ_1‐42_, t‐tau, t‐α‐syn, o‐α‐syn, pSer129‐α‐syn, age, and sex discriminated the DLB group from the controls. Low levels of Aβ_1‐42_ (OR, 0.42; 95% CI, 0.22‐0.80), high levels of tau (OR, 3.62, 95% CI, 1.58‐8.27), low levels of total α‐syn (OR, 0.30; 95%CI: 0.13‐0.74), and high levels of o‐α‐syn (OR, 4.55; 95% CI, 1.78‐11.66) give a higher risk for DLB compared with controls. For the discrimination between DLB and AD, we found that low levels of tau (OR, 0.22; 95% CI, 0.10‐0.50) and high levels of o‐α‐syn (OR, 2.67; 95% CI, 1.03‐6.94) give a higher risk for DLB compared with AD. Finally, low levels of Aβ_1‐42_ (OR, 0.43; 95% CI, 0.22‐0.87) and high levels of tau (OR, 3.63; 95% CI, 1.63‐8.06) give a higher risk for DLB compared with PD. Receiver operating characteristic curves are illustrated in Supplementary Figure 2. All models had an AUC > 0.80.

**Table 2:** Logistic regression analysis of multiple CSF biomarkers

	DLB			
	Predictors	OR for DLB (95% CI)	*P*	Accuracy of model
Controls	Aβ1‐42	0.42 (0.21‐0.77)	< 0.01	AUC: 0.90 (0.84‐0.96) Sens: 68% PPV: 84% Spec: 93% NPV: 85%
	t‐tau	3.61 (1.67‐8.89)	< 0.01	
	t‐α‐syn	0.30 (0.11‐0.68)	< 0.01	
	o‐α‐syn	4.55 (1.91‐12.87)	< 0.01	
	Age	0.91 (0.81‐1.00)	< 0.05	
	Sex	0.19 (0.04‐0.64)	< 0.05	
AD	t‐tau	0.21 (0.09‐0.43)	< 0.001	AUC: 0.84 (0.75‐0.93)
	o‐α‐syn	2.90 (1.24‐7.97)	< 0.05	Sens: 81% PPV: 79% Spec: 74% NPV: 77%
PD	Aβ1‐42	0.43 (0.20‐0.82)	< 0.05	AUC: 0.84 (0.75‐0.93) Sens: 74% PPV: 85% Spec: 88% NPV: 79%
	t‐tau	3.65 (1.76‐8.86)	< 0.01	
	Sex	0.23 (0.05‐0.85)	< 0.05	

CSF biomarker predictors were Z‐transformed before analyses; therefore, odds ratios (ORs) represent higher odds for DLB per standard deviation (SD) decreased amyloid and t‐α‐syn or increased tau and o‐α‐syn.

Aβ1‐42, amyloid‐β1‐42; AD, Alzheimer's disease; α‐syn, α‐synuclein; AUC, area under the curve; DLB, dementia with Lewy bodies; NPV, negative predictive value; o‐α‐syn, α‐synuclein oligomer; OR, odds ratio; PD, Parkinson's disease; PPV, positive predictive value; Sens, sensitivity; Spec, specificity; t‐α‐syn, total α‐synuclein; t‐tau, total tau protein.

## Discussion

The major findings of this study are that CSF levels of t‐α‐syn are lower in DLB and PD patients compared with both AD patients and cognitively normal controls, whereas CSF levels of o‐α‐syn are higher in DLB and PD patients. In addition, we observed that CSF t‐α‐syn was associated with t‐tau and p‐tau, whereas o‐α‐syn was not associated with any of the CSF AD biomarkers. Third, we demonstrated that CSF α‐syn species in combination with the AD biomarkers are promising biomarker candidates for DLB.

Most previous research on CSF α‐syn in DLB patients focused on t‐α‐syn and generated conflicting results compared with AD patients or controls, with α‐syn levels reportedly increased, decreased, or unchanged (see references 13 and 14 for review). These discrepancies are likely because pf differences in the assay platform, antibodies’ characteristics, CSF collection, storage and processing steps, blood contamination and heterogeneity of patients included in the studies.^13^ By using highly specific and sensitive ELISAs^20^ in a well‐characterized cohort of patients with DLB, PD, and AD and nondemented controls, we now report a decrease of t‐α‐syn in DLB and PD patients compared with AD patients and controls. Moreover, we observed elevated levels of o‐α‐syn in both DLB and PD patients, especially compared with the levels in AD patients and controls. These findings are in line with previous CSF studies that showed increased o‐α‐syn levels in DLB patients compared with AD patients^22^ and in PD patients compared with controls.^23^, ^24^, ^25^ However, we did not observe differences in pSer129‐α‐syn levels between diagnostic groups. To date, no previous studies have evaluated CSF pSer129‐α‐syn in a DLB patient cohort. In a previous study in PD patients using a Luminex assay, however, CSF pSer129‐α‐syn levels were increased in PD patients compared with healthy controls, but not compared with AD patients.^27^ In the present study, we observed a trend toward higher CSF levels of pSer129‐α‐syn in PD patients compared with controls as previously reported,^20^ but possibly as a result of the large dispersion of pSer129 within the groups, especially in the control group, this increase did not achieve statistical significance. We found a negative association between pSer129‐α‐syn and MMSE only in DLB patients. These findings together might suggest that pSer129‐α‐syn will not aid in differential diagnosis, but rather that pSer129‐α‐syn might play a role specific in DLB (and PD).

The reduction in t‐α‐syn in DLB and PD patients is likely because of α‐syn aggregation and sequestration in Lewy bodies,^6^ similar to the reduction in CSF Aβ_1‐42_ that is thought to mirror increased amyloid deposition in the AD brain. However, the regulation of t‐α‐syn in DLB seems to be more complex. We observed a positive association between tau proteins and t‐α‐syn in DLB, PD, and AD patients, but not in controls. These results concur with previous studies.^35^, ^36^, ^37^, ^38^, ^39^ Tau protein is considered a biomarker of neurodegeneration.^7^ Synapse loss and disruption could cause a release of tau and t‐α‐syn from damaged neurons into the brain's interstitial fluid and then into the CSF, resulting in higher CSF levels of both tau and t‐α‐syn. Hence, it could be hypothesized that DLB patients with more synaptic loss have elevated levels of t‐α‐syn, whereas DLB patients with limited synaptic loss have decreased levels of t‐α‐syn. This hypothesis is supported by the findings that CSF levels of t‐α‐syn are elevated in AD, characterized by marked neuron and synapse loss, compared with controls^39^ and t‐α‐syn levels increased with disease progression in PD.^40^, ^41^ In the present study, we found a negative correlation (*r* = ‐0.42) between t‐α‐syn and MMSE score in PD patients. This finding is in line with previous studies.^41^, ^42^ Studies with longitudinal measurements of CSF biomarkers in PD indeed showed that t‐α‐syn and tau increased over 2 years in PD patients and were associated with worsening cognition.^40^, ^41^ A possible explanations for the association might be that impaired synaptic function is linked to cognition in Parkinson's disease.^43^, ^44^, ^45^ In line with our results, most studies performing correlation analysis between t‐α‐syn and AD biomarkers showed a positive correlation between t‐α‐syn and tau and no correlation with Aβ_1‐42_ in patients with PD/DLB.^20^, ^25^, ^35^, ^37^, ^46^, ^47^, ^48^, ^49^ However, other studies have shown a positive correlation between t‐α‐syn and Aβ_1‐42_ in patients with PD/PDD.^26^, ^41^, ^50^, ^51^, ^52^ This discrepancy might be a result of inclusion of more severely affected PD patients with lower levels of CSF Aβ_1‐42_. In a previous study in early PD patients, no correlation between t‐α‐syn and Aβ_1‐42_ was found.^53^ These results seem to suggest that t‐α‐syn and Aβ_1‐42_ reflect unrelated disease processes. The elevated levels of o‐α‐syn might be associated with increased levels of soluble α‐syn aggregates resulting from a clearance failure.^54^, ^55^

Although differences in t‐α‐syn and o‐α‐syn were found between diagnostic groups, there is substantial overlap of individual α‐syn levels, which limits the diagnostic value of α‐syn species for individual patients. A potential confounding factor is the overlap in histopathology in neurodegenerative diseases. Neuropathological studies reported the presence of α‐syn pathology in 20%‐50% of AD patients.^36^, ^56^, ^57^, ^58^ In addition, α‐syn pathology was also present in approximately 25% of aged healthy controls.^59^ Another possible explanation could be that these CSF α‐syn species are not sensitive or disease specific enough to distinguish DLB and/or PD from AD and controls. Several authors have suggested that CSF α‐syn species might be more informative when used in combination with other biomarkers, for example, Aβ_1‐42_, t‐tau, and p‐tau.^25^, ^36^, ^37^, ^49^ In the present study, we demonstrated that CSF α‐syn species add discriminatory value to traditional CSF AD biomarkers. AD biomarkers can be used to discriminate both types of dementia (ie, AD and DLB) from PD and controls, and α‐syn species add by discriminating both types of α‐synucleinopathies (ie, PD and DLB) from AD and controls, illustrating that DLB is at the crossroads of dementia disorders and α‐synucleinopathies. This was further substantiated when we found that in a bilateral comparison, the combination of o‐α‐syn and tau optimally discriminates DLB from AD. Taken together with the results of previous studies,^38^, ^46^, ^49^, ^60^ our observations underline the potential of combining α‐syn species with other biomarkers like Aβ_1‐42_, tau, and p‐tau to improve the differential diagnosis of DLB. Other, yet to be discovered potential biomarker candidates or posttranslationally modified α‐syn species, may also be useful for this purpose.

One of the strengths of this study is that the diagnosis of PD and DLB was supported by ^123^I–FP‐CIT SPECT findings showing presynaptic dopaminergic deficits and/or slow‐wave activity on EEG. Furthermore, the cohort was relatively large for a CSF biomarker study. Third, our assays are sensitive, highly target specific, and robust. Among the limitations is the lack of postmortem validation in most patients. Only one DLB patient underwent postmortem examination. Another limitation is the use of erythrocytes instead of hemoglobin to measure the contamination of red blood cells in CSF. The erythrocytes were measured in the first 2 mL of CSF during routine analysis and might not reflect the actual erythrocyte count in the CSF sample used to measure α‐syn species. Using this procedure we may have overestimated the actual erythrocyte count. To note, as we excluded all CSF samples with an erythrocyte count ≥ 500 cells/μL, it is unlikely that traces of blood may have influenced CSF α‐syn levels in our study.

In conclusion, DLB is a disease entity that is located at the crossroads of dementia disorders and movement disorders. We here shown that CSF α‐syn species, especially t‐α‐syn and o‐α‐syn, in combination with the AD biomarkers could be useful as part of a biomarker panel to support DLB diagnosis. This approach would allow for better and timelier diagnosis, characterization of disease subtypes, patient selection for clinical trials that are designed to evaluate new disease‐modifying treatments, and treatment monitoring. An important next step is to prospectively validate CSF α‐syn species in patients at an early disease stage or in a prodromal phase.

## Author roles

Inger van Steenoven: 1B, 1C, 2A, 2B, 3A.

Nour Majbour: 1C, 3B.

Nishant Vaikath: 1C.

Henk Berendse: 2C, 3B.

Wiesje van der Flier: 2A, 2C, 3A, 3B.

Wilma van de Berg: 1A, 1B, 2C, 3B.

Charlotte Teunissen: 1A, 1B, 2C, 3A, 3B.

Afina Lemstra: 1A, 1B, 2C, 3A, 3B.

Omar El‐Agnaf: 1A, 1B, 2C, 3B.

## Financial disclosures

Dr. Teunissen serves on the advisory board of Fuijirebio and Roche, received research consumables from Euroimmun, IBL, Fujirbio, Invitrogen and Mesoscale Discovery, and performed contract research for IBL, Shire, Boehringer, Roche and Probiodrug; and received grants from the European Commission, The Dutch Research Counsil (ZonMW), Association of Frontotemporal Dementia/Alzheimer's Drug Discovery Foundation, ISAO and the Alzheimer's Drug Discovery Foundation.

Dr. van der Flier performs contract research for Biogen MA Inc. Research programs of dr. van der Flier have been funded by ZonMW, NOW, EU‐FP7, EU‐JPND, Alheimer Nederland, Cardiovasculair Onderzoek Nederland, stichting Dioraphte, Gieskes Strijbis fonds, Boehringer Ingelheim, Piramal Neuroimaging, Roche BV, Janssen Stellar, and Combinostics. All funding is paid to her institution.

The remaining authors report no actual or potential conflicts of interest.

## Supporting information

Supporting Information
