# Supplementary material for: α‐Synuclein species as potential cerebrospinal fluid biomarkers for dementia with lewy bodies
Source: Mov Disord. 2018 Nov 15;33(11):1724–33. doi: 10.1002/mds.111 (PMC6519232; doi:10.1002/mds.111)
Supplement: Supplementary file 1 — Supporting Information [file MDS-33-1724-s001.pdf]

Supporting Information

Supplementary table 1. Characteristics of the control groups

	SCD (n=30)	Healthy controls (n=48)	p-value
MMSE<br>median [IQR]	29 [28-30]	29 [29-30]	0.611
t-α-syn (ng/ml)<br>mean ± SD	1.8 ± 0.6	1.9 ± 0.8	0.854
o-α-syn (pg/ml)<br>mean ± SD	82 ± 29	68 ± 41	0.169
pSer129-α-syn (pg/ml)<br>mean ± SD	304 ± 184	227 ± 55	0.152

Data are expressed as mean ± SD or median [interquartile range].
Differences between groups were assessed with student t-tests for normally distributed continuous variables or with Mann-Whitney U tests for non-normally distributed continuous variables.
MMSE = Mini-mental state examination; pSer129-α-syn = phosphorylated α-synuclein protein at Serine 129; o-α-syn= α-synuclein oligomers; SCD = Subjective cognitive decline; t-α-syn = total α-synuclein

Supplementary table 2. Characteristics of outliers

Outliers ID	Diagnosis	Age	Sex	t-α-syn (ng/ml)	o-α-syn (pg/ml)	pSer129-α-syn (pg/ml)	Aβ1-42 (pg/ml)	t-tau (pg/ml)	p-tau (pg/ml)
ADC-5085	DLB	67.8	f	1.23	161	**798**	484	557	66
ADC-3709	DLB	76.5	m	1.58	138	**697**	912	218	34
ADC-4081	DLB	73.3	f	0.72	86	**627**	617	134	17
MOV-18	PD	84.0	m	1.32	**343**	197	431	116	51
MOV-94	PD	59.0	m	2.02	**299**	253	1234	393	73
ADC-223	AD	66.4	m	**5.55**	95	196	237	425	55
ADC-3416	AD	55.9	m	2.49	139	**639**	460	921	99
ADC-3418	AD	65.0	m	3.05	58	**600**	634	620	75
MOV-176	HC	79.0	m	**4.58**	56	164	1414	458	63
ADC-3490	SCD	62.2	m	2.70	97	**968**	1280	300	47
ADC-1157	SCD	66.2	m	1.60	106	**713**	848	188	71
ADC-158	SCD	70.0	f	1.28	100	**676**	192	251	44

Values in bold are above the third quartile plus 3xIQR
Aβ1-42 = amyloid β1-42; AD = Alzheimer's disease; DLB = Dementia with Lewy bodies; f = female; m = male; PD = Parkinson's disease; pSer129-α-syn = phosphorylated α-synuclein protein at Serine 129; p-tau = tau phosphorylated at threonine 181; o-α-syn= α-synuclein oligomers; SCD = Subjective cognitive decline; t-α-syn = total α-synuclein; t-tau = total tau

Supplementary table 3. Associations between CSF biomarkers

	t-α-syn	o-α-syn	pS129-α-syn	Aβ1-42	t-tau	p-tau
t-α-syn	1					
DLB		- 0.31	- 0.13	- 0.03	0.48**	0.40*
PD		0.09	- 0.05	0.14	0.64***	0.69***
AD		0.08	0.16	- 0.34	0.45*	0.44*
Controls		- 0.16	- 0.16	0.07	0.17	0.20
o-α-syn		1				
DLB	- 0.31		0.45*	0.03	0.00	- 0.03
PD	0.09		- 0.08	- 0.01	- 0.11	- 0.04
AD	0.08		0.24	0.10	0.30	0.30
Controls	- 0.16		0.02	- 0.02	- 0.05	- 0.02
pSer129-α-syn			1			
DLB	- 0.13	0.45*		0.06	0.18	0.02
PD	- 0.05	- 0.08		- 0.11	- 0.14	- 0.29
AD	0.16	0.24		0.23	0.23	0.36
Controls	- 0.16	0.02		- 0.26	- 0.03	- 0.05

Associations between CSF biomarkers were assessed with Pearson Correlation Coefficients. The Benjamini-Hochberg procedure was used to correct for multiple testing. Data shown as r. Significance: *** p<0.001; ** p<0.01; * p<0.05

Aβ1-42 = amyloid β1-42; AD = Alzheimer's disease; DLB= Dementia with Lewy bodies; PD= Parkinson's disease; pSer129-α-syn= phosphorylated α-synuclein protein at Serine 129; p-tau = tau phosphorylated at threonine 181; o-α-syn= α-synuclein oligomers; t-α-syn= total α-synuclein; t-tau = total tau

Supplementary table 4. Associations between CSF α-syn species and clinical parameters

	Age	Disease duration	MMSE	UPDRS-III
t-α-syn				
DLB	0.11	0.18	0.30	NA
PD	0.17	0.22	- 0.42**	- 0.06
AD	- 0.07	0.10	0.09	NA
Controls	0.06	NA	0.10	NA
o-α-syn				
DLB	0.12	- 0.04	- 0.10	NA
PD	0.09	- 0.28	- 0.02	0.10
AD	0.16	0.04	0.33	NA
Controls	0.16	NA	- 0.11	NA
pSer129-α-syn				
DLB	0.39*	- 0.21	- 0.45**	NA
PD	- 0.15	- 0.10	0.16	- 0.24
AD	- 0.28	0.11	- 0.05	NA
Controls	0.04	NA	- 0.03	NA

Associations between CSF α-syn species and clinical variables were assessed with Pearson Correlation Coefficients. Data shown as r.

Significance: *** p<0.001; ** p<0.01; * p<0.05

AD = Alzheimer's disease; DLB= Dementia with Lewy bodies; MMSE= Mini-mental state examination; NA = not applicable; UPDRS-III = Unified Parkinson Disease Rating Scale motor score; PD= Parkinson's disease; pSer129-α-syn= phosphorylated α-synuclein protein at Serine 129; o-α-syn= α-synuclein oligomers; t-α-syn= total α-synuclein

Supplementary table 5. Discriminant loadings for each individual predictor

	Function	
	1	2
Aβ1-42	**- 0.64**	- 0.11
t-tau	**0.81**	- 0.19
t-α-syn	0.19	**- 0.62**
o-α-syn	0.01	**0.83**

The correlation coefficient represents the relative contribution for each predictor to group separation.

Aβ1-42 = amyloid β1-42; o-α-syn= α-synuclein oligomers; t-α-syn= total α-synuclein; t-tau = total tau

Supplementary table 6. Logistic Regression analysis of multiple CSF biomarkers for discrimination between PD and controls

	**PD**				
	**Predictors**	**OR for PD(95% CI)**	**p-value**	**Accuracy of model**	
**Controls**	t-α-syn	0.27 (0.11-0.57)	<0.01	AUC: 0.85 (0.77-0.92)	
	o-α-syn	3.32 (1.97-6.09)	<0.001	Sens: 67%	PPV: 74%
				Spec: 87%	NPV: 82%

CSF biomarker predictors were Z transformed before analyses; therefore, odds ratio's (OR) represent higher odds for PD per standard deviation (SD) decreased t-α-syn or increased o-α-syn.

α-syn = α-synuclein; AUC = area under the curve; NPV = Negative predictive value; o-α-syn = α-synuclein oligomers; OR = odds ratio; PD = Parkinson's disease; PPV = positive predictive value; Sens = Sensitivity; Spec = Specificity; t-α-syn = total α-synuclein.

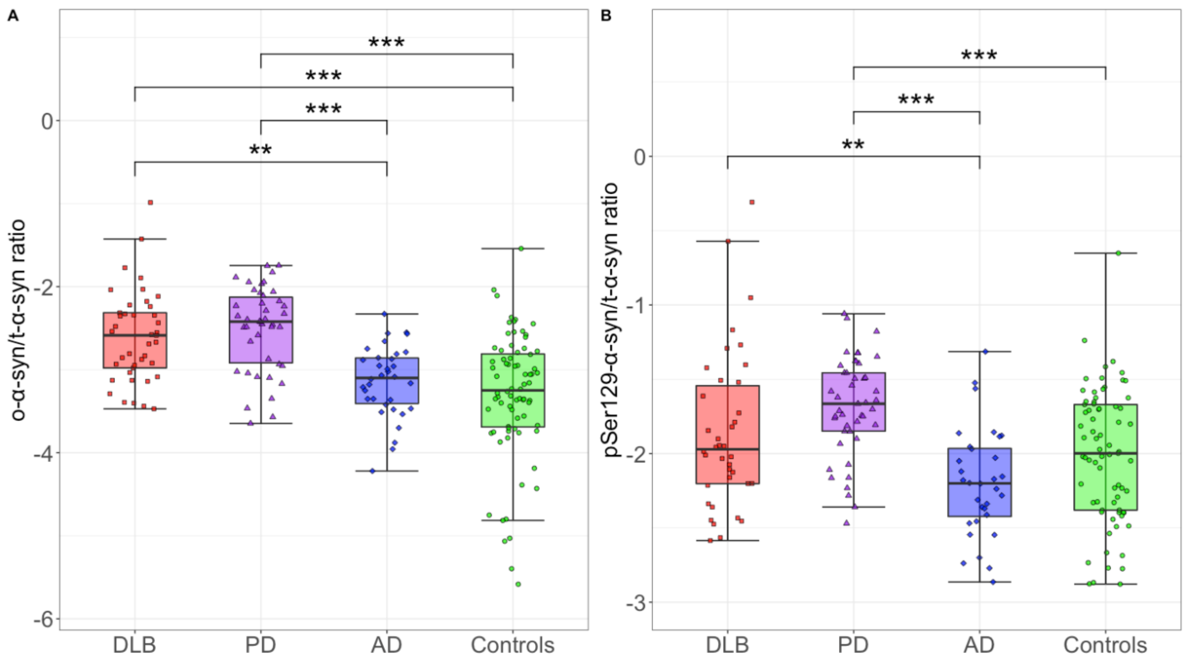

Supplementary figure 1. Box and Whisker plots of ratio's of α-syn species in DLB, PD, AD and controls

(A) Ratio of o-α-syn/ t-α-syn, (B) Ratio of pSer129-α-syn/ t-α-syn. The line through the middle of the boxes corresponds to the median and the lower and the upper lines to the 25th and 75th percentile, respectively. The whiskers extend from the 5th percentile on the bottom to the 95th percentile on top. Differences between groups were assessed with GLM, adjusted for age and gender.

* p<0.05, ** p<0.01, *** p<0.001

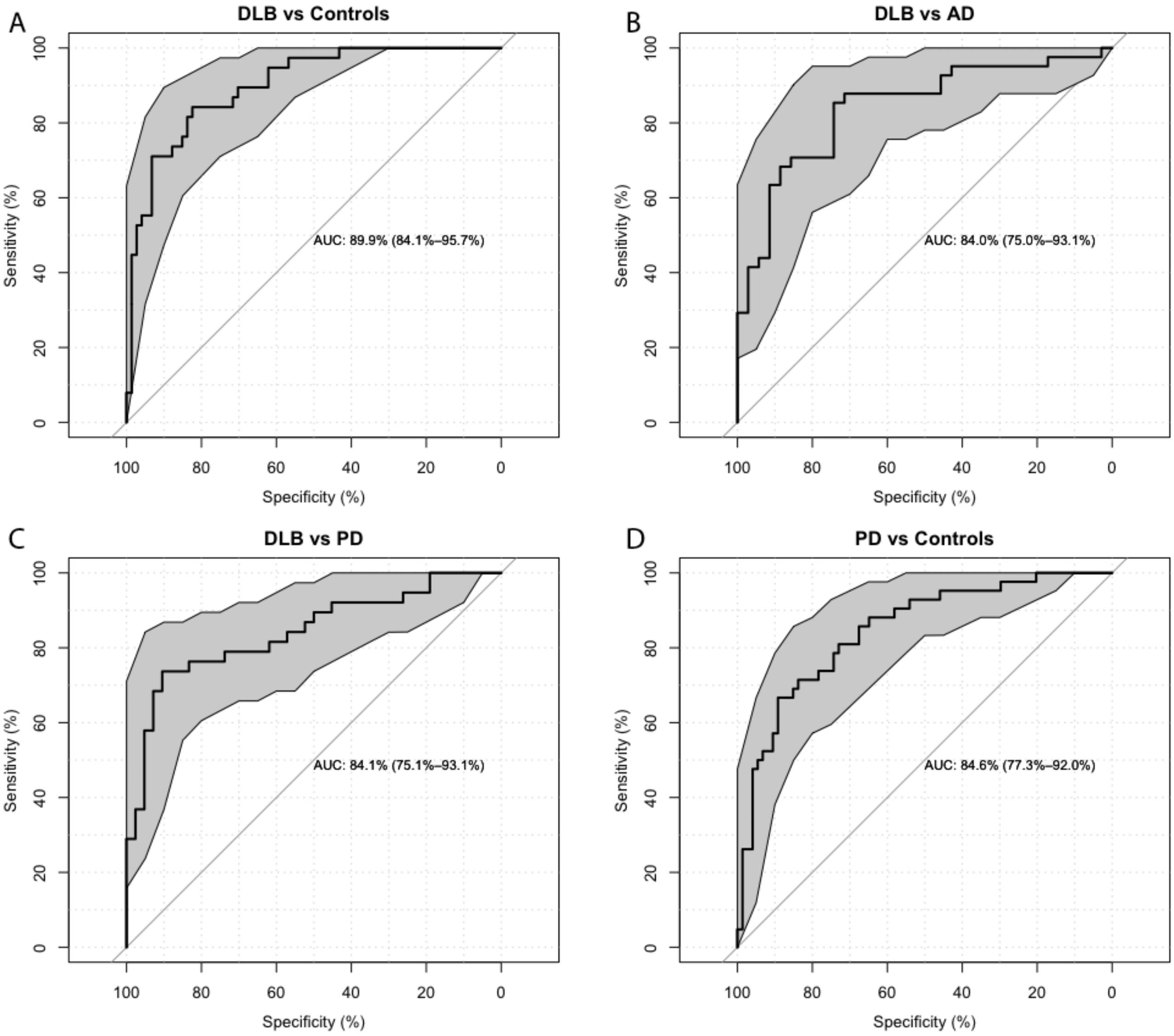

Supplementary figure 2. Receiver operating characteristic curves (ROC) showing the diagnostic accuracy of the final logistic regression models

(A) DLB vs Controls, (B) DLB vs AD, (C) DLB vs PD, (D) PD vs Controls. The grey area represent the 95% confidence intervals.

Supporting information: Immunoassays to quantify α-syn species in the CSF.

For measuring CSF t-α-syn, pSer129-α-syn or o-α-syn, a 384-well ELISA microplate was coated by overnight incubation at 4°C with 0.1 µg/ml Syn-140 (sheep anti-α-syn polyclonal antibody), 0.1 µg/ml Syn-140 or 0.2 µg/ml Syn-O2 (mouse oligomeric specific α-syn mAb) respectively. Following blocking non-specific binding using 2.5% gelatin in PBST, samples were added to corresponding wells and plate was incubated. As reporter antibodies, 11D12 (mouse anti-α-syn monoclonal antibody), PSer129 (mouse anti-pS129-α-syn monoclonal antibody) or and FL-140 (rabbit polyclonal antibody, Santa Cruz Biotechnology, Santa Cruz, CA, USA) were used for measuring t-α-syn, pSer129-α-syn or o-α-syn, respectively. The t-α-syn capture antibody (Syn-140) and t-α-syn detection antibody (11D12) can detect different forms of α-syn (nitrated, phosphorylated, oligomeric and monomeric). Therefore, our t-α-syn ELISA measures wide range of α-syn species, with no preference for the monomeric form. Following the incubation with species-appropriate secondary antibodies (donkey anti-mouse IgG HRP or goat anti-rabbit IgG HRP, Jackson ImmunoResearch Lab- oratories Inc., USA), plates were incubated with enhanced chemiluminescent substrate (SuperSignal ELISA Femto, Pierce Biotechnology, Rockford, Illinois, US). The chemilluminescence, expressed in relative light units, was immediately measured using Perkin Elmer Envision plate reader. The lower limit of detection of the t-α-syn assay, o-α-syn assay and pS129-α-syn assay were 50 pg/mL, 10 pg/mL and 20 pg/mL, respectively. A series of internal controls was run to check for run-to-run variations. The inter-assay coefficient of variation for t-α-syn, o-α-syn and pSer129- α-syn were between 3% and 5.5%. The intra-assay coefficient of variations were <10%, calculated by averaging the coefficient of variation of duplicates.
